# A Brief History of Bioethics in Japan

**DOI:** 10.1007/978-981-15-3572-7_1

**Published:** 2020-05-20

**Authors:** Akira Akabayashi

**Affiliations:** Department of Biomedical Ethics, University of Tokyo, Faculty of Medicine, Tokyo, Japan

## Abstract

In this chapter I look back at the history of bioethics in Japan, which can be divided into three phases: Phase I, Introduction (1980–1999); Phase II, Development (2000–2010); and Phase III, the Recent Past (2011–present). Phase I marks the period when the concept of bioethics that originated in the West came to Japan. It was also when Japanese society faced its first difficult bioethical issues: namely brain-death and organ transplantation. Other issues emerged during this period, particularly pertaining to death, such as end-of-life medical care and euthanasia. In Phase II, the problems shifted to those pertaining to the beginning of life, such as the moral status of the human embryo. As well, during this period the government implemented ethical guidelines for research ethics. During this period, social awareness of bioethics increased, and bioethics education began to appear not only medical education, but also within high school curricula. In Phase III, Japan began to tackle its own ethical issues, such as enhancement, regenerative medicine, neuroethics, public health ethics, and precision medicine. Some of my thoughts concerning projections for the future are discussed at the end of this chapter.

In this introductory chapter, I consider the current period (ca. 2020), look back on the history of bioethics in Japan over the past 40 years, and finally, look briefly toward the future. First, I present an overview of the types of problems that have been handled in the fields of bioethics and medical ethics in Japan since the 1980s. I begin my discussion in this era because modern bioethics began its development in Japan in the early 1980s. I divide this time period (beginning in the early 1980s until around 2020) into three parts, corresponding to Phase I, Introduction: 1980–1999; Phase II, Development: 2000–2010; and Phase III: the Recent Past (2011-present). No significant distinction is intended between use of the terms “bioethics” and “medical ethics.” For full description, refer to references [1–3]. This chapter is a brief summary of those three papers and some further considerations.

## Phase I: Introduction (1980–1999)

Bioethics is said to have been born in the USA in the 1960s. In the early 1980s in Japan, bioethics literature was introduced and texts were translated from English, primarily at universities and other academic institutions. My own interest in issues such as euthanasia, dying with dignity, disclosing a cancer diagnosis, abortion, and genetic manipulation developed when I was a medical student in the 1970s. I did my best to learn about these subjects, but at the time, Japan had no academic field equipped to handle these types of problems. While attending an exchange event at the Japan-America Student Conference, an American student informed me, “You are interested in a field called bioethics.” This was the first time I heard the term “bioethics.” In 1979, Beauchamp and Childress published their first edition of the *Principles of Biomedical Ethics*; this was concurrent with bioethics being established as an academic field in the USA.

### Brain-Death and Organ Transplantation

In Japan, the issue of brain-death and organ transplantation was highly influential to the development of bioethics (see Chap. 10.1007/978-981-15-3572-7_2). Beginning in the 1980s, nationwide debates began as to whether brain-death constituted human death, and whether organ transplantation from a brain-dead body should be permissible. These issues were discussed in medical circles, religious groups, political groups, the media, and among the general population. With the establishment of a commission on brain-death, and the 1997 enactment of the Organ Transplantation Law, organ transplantation from a brain-dead body was finally deemed permissible, with strict conditions. Specifically, the clear and written expression of the intent to donate from the organ donor (15 years or older) as well as the family’s consent were both required. Politically, this marked a major milestone. Several positive outcomes were achieved, including discussion on the definition of death and Japanese views on life and death; this set a healthy tone for how bioethical arguments should be handled in Japan from that point forward.

### Informed Consent

At about the same time, the idea of informed consent began to emerge as an issue in clinical settings (see Chap. 10.1007/978-981-15-3572-7_3). Informed consent in the clinical context entails an act in which “medical caregivers provide sufficient explanation to those with sound judgment capacity and ensure that the patient understands, while the patient then offers consent of their own volition.” Today this is considered common protocol, but such was not always the case in Japan. There was even extensive debate on how to translate the English term “informed consent” into Japanese; today, the term “*info-mudo konsento*” is used, but is presented in *katakana*, the phonetic alphabet used for foreign terms and names. In the course of this discussion, those arguing to protect patient autonomy, basing their arguments on self-determination and the right to know, were in a dominant position. Others countered that argument, stating that prioritizing individual autonomy so heavily is unsuited for Japan. However, an explanation of the patient’s condition has now been systematically added to the treatment plan at the time of hospital admission and discharge, such that it is covered by health insurance [4]. Informed consent is therefore a common theme that has been integrated into medical care. This flow of events necessitated a change in paternalism among physicians. It also led to the medical record disclosure system based on the Private Information Protection Law in 2003.

### Issues with End-of-Life Medical Care and Euthanasia

One other major event during Phase I was the 1991 Tokai University Euthanasia Incident. In this case, a physician, in response to a clear request from the patient’s family, administered potassium chloride (KCl) to a terminal cancer patient [5]. He was found guilty of murder and given a suspended sentence by the Yokohama District Court in 1995. The trial was an unprecedented case in which a doctor performed euthanasia. As the court decision presented the conditions that would allow for active euthanasia in the obiter dictum, it was erroneously relayed to other countries that active euthanasia was legally permissible in Japan. Reports of physician-assisted suicide emerged from abroad, and terms such as euthanasia, mercy killing, and dying with dignity were used without clarity. Currently in Japan, the act of a medical caregiver administering muscle relaxants or KCl to a patient, leading to the latter’s death, carries with it a high probability that the medical caregiver will be placed on trial for murder. Notably, unlike several other countries, Japan has not legalized active voluntary euthanasia. In addition, with the increased popularity of hospice or palliative care, fewer cases of “withholding treatment” requested ahead of time by patients are legally problematic. However, the issue of “treatment withdrawal” (removal of an artificial respirator) from a terminal patient has yet to be subject to legal resolution (see Chap. 10.1007/978-981-15-3572-7_4).

## Phase II: Development (2000–2010)

There are three characteristics of Phase II. First, the topics of discussion shifted from issues pertaining to the end of life (brain-death, end-of-life medical care, and euthanasia) to those dealing with the beginning of life, particularly the moral status of the embryo and problems related to reproductive medicine. Second, numerous policy guidelines and legislation were produced in the fields of life sciences and medical care, paving the way for the establishment of a framework for policy decisions. Third, bioethics came to be socially acknowledged in the fields of medical education and research.

### On the Moral Status of the Embryo

In the second phase, topics in medical ethics shifted to issues pertaining to the beginning of life. Among them, the heightened discussion surrounding the moral status of the human embryo became particularly significant. At this time, research on embryonic stem cells (hereafter, ESCs) had become widespread. Human ESCs have the capacity to differentiate into all kind of tissues and all cell types, and there was great hope for these cells in the field of regenerative medicine. However, in order to create human ESCs, one must destroy a fertilized egg (embryo) that has the capacity to become a human individual in the future. If one perceives that human life is present in an embryo, then this type of use of embryos cannot be approved. In 2004, the Council for Science and Technology Policy and the Experts Panel on Bioethics compiled the document “Basic Principles Concerning the Handling of Human Embryos.” This document stated that the human embryo is not a person per se, but it is also not an object. The document instead uses the phrase, “sprout of human life.” The writers conclude that a human embryo is worthy of respect and requires careful handling (see Chap. 10.1007/978-981-15-3572-7_5). Fundamentally, “The Guidelines for Derivation and Utilization of Human Embryonic Stem Cells” and “The Act on Regulation of Human Cloning Techniques” take the same stance as “The Basic Principles Concerning the Handling of Human Embryos.” Given that the moral status of the human embryo is a topic of deep religious and political debate in other countries, it seems that Japan has actually handled this issue with relative ease. However, it remains a mystery as to why abortion—another debate that should be begging many questions about the moral status of an individual prior to birth—did not gain as much momentum as that surrounding the human embryo.

### Systematization of the Enactment Processes for the Life Sciences and Medical Care

At the end of the twentieth century, numerous governmental ethical guidelines (Ethical guidelines for human genome/gene analysis research, Ethical guidelines for epidemiological research, and Ethical guidelines for clinical research) as well as a number of laws (The Organ Transplant Law, The Act on Regulation of Human Cloning Techniques) came into the fields of life science and medical care. Many of these were drafted by governmental review boards or committees. These drafting sessions came to include medical and legal specialists as well as ethicists, and representatives from the general public such as journalists. By this time, a new framework had been put in place for policy-making in the fields of life sciences and medical care. This was a linear process that began with forming a public committee comprising specialists from many disciplines, drafting the guidelines, implementing the public comment system, and publishing the final results on the internet, followed by media reporting and revisions over several years. This framework reflected the nature and process of the debate surrounding organ transplantation from brain-dead donors.

### Ethics Education in Medicine and in Research

By the year 2000, “Medical Ethics” had been added to the curriculum of medical schools nationwide, and faculty members were tasked with teaching these classes. In the early stages, these classes were taught by faculty in forensic medicine, public health, and philosophy/ethics departments as part of general education courses. However, in 2000, the first graduate level Department of Biomedical Ethics was established at the Kyoto University Graduate School of Medicine, and several full-time faculty members were hired. In 2003, the University of Tokyo Graduate School of Medicine also created a Department of Biomedical Ethics. As this process continued to unfold, the “ethics of medicine” education for students in healthcare-related areas became standard. Meanwhile, the ELSI (Ethical, Legal, and Social Implications) programs in the life sciences and medicine were offered a substantial amount of public and private research funding. By the end of Phase II, bioethics had earned a well-respected place in education and research.

## Phase III: The Recent Past (2011–Present)

In what follows I introduce several topics that have emerged or are just emerging in Japan. These are not unique to Japan, but I would like to emphasize those which have become prominent in the Japanese context.

### Enhancement

“Enhancement” refers to a specific use of medical technology, that is not been used to treat or prevent disease. It has been defined as an “intervention aimed to improve the human form and function more than is necessary for maintaining health or recovery” [6]. A more straightforward definition is set forth by Kato, a Japanese philosopher, who defines enhancement as “employing medical technology for a purpose other than treatment.” Examples of enhancement include the administration of growth hormones for children who are not suffering from growth restriction, “doping” or the use of steroids to enhance muscle strength in sports, and the use of mind-altering drugs such as Ritalin (methylphenidate) and Prozac (fluoxetine hydrochloride) by healthy individuals to enhance their attention (learning capacity) or mood (“smart pills” or “happy pills”). In the future, we may be dealing with designer babies due to genome editing, increased longevity, and enhancement using a brain–machine interface that can operate machinery by connecting a brain to a computer.

Ideas to improve humans are not new, but developments in medical technology now present the possibility of altering the human body, which has led to many debates surrounding the ethical issues involved. Those opposed to enhancement are concerned about its widespread use, advocating that it is unnatural for human beings to “play God,” that there are unknown dangers, and that we would lose sacred values such as the importance of weakness and being interlinked with one another. Meanwhile, those whose stance is one of the passive promotion, that is “so long as each individual’s freedom to choose is respected we cannot go so far as to prohibit this,” are in favor of enhancement, as are those who actively promote it based on utilitarian principles, arguing that enhancement promotes the happiness and enjoyment of human beings, and thus we are obligated to pursue it.

In Japan, rather than either approving or prohibiting all forms of enhancement, each issue is considered individually. Some also feel that as long as society respects individual self-determination and the multidimensionality of values, blanket prohibition of enhancement cannot be enforced, but some restrictions may be warranted. These discussions will help us to grapple with some important issues related to the nature of human beings and society.

### Neuroethics

In recent years, a new and rapidly developing field of study has emerged that deals with ethical problems related to neurology or neuroscience and applicable technologies. This field has come to be known as “neuroethics.” Safire defines neuroethics as “the examination of what is right and wrong, good and bad about the treatment of, perfection of, or unwelcome invasion of and worrisome manipulation of the human brain” [7]. Two major developments in neuroscience aided in creating momentum for neuroethics. First, the development of brain functional imaging technology such as PET and fMRI made it possible to observe the brain functions of a living human being. This new technology presents the possibility of obtaining an even more diverse array of information, such as lie detection and clarifying an individual’s level of consciousness. However, it also opens up possibilities for mind-reading, or the ability to read the condition of another person’s spirit. The second major technological development involves selective pharmacological and anatomical intervention/manipulation of neurological processes. For example, it is now possible to control tremors caused by Parkinson’s disease with Deep Brain Stimulation. This technology could potentially be applied to patients with other diseases. Moreover, it could also be used for brain enhancement in healthy people. Brainwashing and mind control for military purposes have also come into the debate.

The thought of a brain–machine interface and chimeras of humans and animals being created evokes science fiction-like images of cyborg production, generating fear among people. Some attempts have been made to resolve this fear through two-way communication between scientists and non-scientists, for example, and by improving scientific literacy among the general population and performing risk assessments and encouraging participation from ordinary citizens. The Japanese government is proactive about neuroscience research and has a national brain science project; the Strategic Research Program for Brain Science. While there are no specific topics unique to Japan, ELSI studies of techniques developed in Japan such as decoded neurofeedback are ongoing.

### Ethical Issues Surrounding Regenerative Medicine

In 2012, Dr. Shinya Yamanaka created the Nobel Prize-winning human induced pluripotent (iPS) cells at Kyoto University, using technology that allows for the creation of cells with the same differentiation function as human ESCs by incorporating multiple genes into an adult somatic cell. This technology did not require the destruction of a human embryo, and thus generated much hope for cell transplantation medicine that does not involve ethical issues or rejection responses. It was a breakthrough in the field of life sciences as well as the ethical arena. Currently, iPS cell research has moved from the basic research stage to drug development and even clinical applications such as cell transplantation. The Japanese government is actively promoting iPS cell research. However, were the ethical problems truly and completely eliminated by iPS cell technology? Here, I discuss some of the remaining issues.

The first of these is safety. The fact that these cells can differentiate into many cell types means that the possibility of cancerization cannot be ruled out. In this regard, attempts are being made to create iPS cells (using drugs, for example) that lower the number of, if not eliminate the need for, gene recombinants. This is something that future technological advancements will likely be able to overcome.

Next, whether or not it is permissible to create reproductive cells (sperm or egg cells) from iPS cells is an important ethical issue. In research using human cloned embryos, there was some concern that if the cloned embryo was returned to the uterus, then an individual (a cloned human being) would develop. As such, returning a cloned embryo to the uterus was prohibited by the Act on Regulation of Human Cloning Techniques. For iPS cell research, at least in theory, if it is possible to induce differentiation into sperm and egg cells, then it may also become possible to fertilize these. As was the case with the argument surrounding cloning, problems arise concerning the uniqueness of a human individual.

Another developing discussion in this field concerns differentiation into neurons. For example, if a human neuron is grown for research in an animal brain, there is some concern that this animal will develop a human personality. This may present to us a problem related to fusion between humans and animals at the cellular or genomic level. Another challenge is that of stem cell research, for which various uses can be imagined (for example, controlling the direction of differentiation, genetic recombination, and fusion with cells from other species). The problem for researchers then becomes how much consent to obtain from cell donors, and for cell donors, how much they should understand about the future destinations of their cells.

### Public Health Ethics

The course of medical ethics in the second half of the twentieth century can be summed up by the phrase “from paternalism to individual self-determination.” Part of the background for this was the change in disease patterns. Namely, from infectious diseases to lifestyle diseases, which led to a change in thinking about disease as something that should be prevented at a population level, to that which should be prevented and treated individually. However, in recent years, the dangers of new and re-emerging infectious diseases such as HIV/AIDS, malaria, SARS, and novel forms of influenza have increased. In addition, concern has mounted for bioterrorism-led smallpox outbreaks, or a recurrence of polio, leading us to refocus our attention on the importance of mass prevention. In Japan, the worldwide spread of the H1N1 influenza virus in 2009 is a recent collective memory. This engendered social debate, especially with regard to who would be the first to receive the vaccine.

It is clear that situations are emerging that cannot be well addressed by the kind of bioethics that prioritizes individual self-determination. The opposition and tension between public welfare and individual freedom can be seen in the debates about quarantines and vaccinations for infectious diseases, discussion on handling vaccine distribution, and individual lives being affected by governmental interventions aimed to promote health. Meanwhile, they also create more difficulties than ever before for equal distribution (justice) of benefit and cost. Simply stated, the central arguments concerning these issues pose the following questions: in what situations should individual autonomy and self-determination be restricted, and what degree of intervention for the sake of the common good and paternalistic interventions should be permitted? A new discussion framework for medical ethics—one that is not limited to the conventional patient–caregiver relationship but rather forms the basis for healthcare policy for the various issues plaguing modern-day public health—is in the process of development.

### Precision Medicine

Discussion concerning this topic is just beginning in Japan. Due to groundbreaking developments in genomic medicine, the time will come when at the time of our birth (or indeed at conception), we will know our genetic destiny. This raises many serious ethical issues. For example, if it is discovered at birth that a child would develop a severe disease by the age of 10, what sort of care should that child receive? What would we do if we found out that we would develop dementia by the age of 50? Would we be tested early on and take preventive drugs? Adults may have the capacity to make these sorts of decisions, but the ethics of administering these tests to underage individuals is not as clear. Is testing justifiable in children if the intent is to prevent disease or slow its progression? Should genome editing be conducted, ensuring that the genes causing disease are eliminated prior to birth? Alternatively, is this an area into which humans should not intervene? This would change the nature of medicine entirely.

Similar issues are raised by the development of artificial intelligence, as implementation of this technology may change the physician’s role markedly. Currently, in an age in which progress has been realized in genomic medicine, precision medicine will likely become one of the most difficult ethical dilemmas for mid-twenty-first century medical care.

## The Future of Bioethics in Japan

Having touched on the history of bioethics in Japan, I will turn to some issues for the future. First, I anticipate that the life sciences and medical technology will continue to progress further, giving hope to many suffering from currently incurable diseases. Some of the developments, such as immune-checkpoint inhibitors, iPS cells, and ESCs, are technological innovations that may help to overcome conventional ethical problems. However, as is evident in issues related to enhancement and neuroethics, forward progress in medical technology that hides within itself great possibilities can, on the one hand, give us great hope for the development of new treatments, while also creating friction between conventional values and ways of living, causing anxiety in society. Moreover, when technology is first introduced, it is difficult to envision sufficiently the influence it will have. How should society handle the “uncertainties” that inevitably and constantly accompany these new technologies? Constructing a framework to ensure that these are addressed effectively is one major challenge for the future of medical ethics in the Japanese context. In addition, even if the arguments in medical ethics shift over to those concerning the beginning of life against the backdrop of new technological developments, issues with terminal medical care and end of life will continue to cause worry among patients and their families, as well as among medical caregivers involved in treatment.

With regard to the many problems that will inevitably emerge, as well as for those that already exist, attempts to address these will likely occur by establishing guidelines or legislation through Japanese governmental committees, as described above. Thus, the framework required to address these issues, while befitting the Japanese context, also comprises an effective method that listens to external voices and adjusts to create harmony in society in order to resolve ethical issues in life sciences and medical care. Yet, I cannot help but assume that the excessive use of guidelines and laws creates a dependency among medical caregivers and researchers, such that they lose the motivation to face problems head-on and think deeply about them, or when necessary, communicate to the outside world about issues.

In questioning whether a hospital ethics consultation (a system in which an ethics committee or its equivalent would offer advice for individual cases) would even function well in this particular climate, I can imagine numerous medical caregivers who would be baffled by having to find answers to difficult ethical issues in the clinical setting. Indeed, the demand not only to accomplish all they do in their busy workday but also to tackle these ethical issues is a tall order. However, we cannot assume simply that because laws and guidelines are in place, all problems will be resolved quickly. If we hope to resolve these ethical issues, we must take a multifaceted approach that is both policy-based (guidelines are issued, ethics committees are established, and healthcare systems are improved) and education-based (awareness and problem-solving capacities are increased among medical caregivers and researchers in those fields as a result of medical ethics education). This is what I envision for bioethics in Japan.

## Before Moving on to the Main Chapters

In the chapters that follow, there are many passages in which I question the Japanese government’s policies in regard to bioethics and research ethics. However, my intent is not to criticize the government, but rather to bring the issues to light and describe them in their rawness through the lens of Japanese culture. If I do not do this, my concern is that international trust and confidence in Japan’s research and medical care will deteriorate over time, and the value quality of Japan as a nation will decrease. This is not a lack of patriotism. As noted in the Introduction, the present book was written to achieve a global scale of bioethical dialogue; to this end, I cannot shy away from criticism of my own country’s policies or cultures in this process when this is necessary.

The late Japanese scholar Donald Keene, in dialogue with Jakucho Setouchi in the book “*Nihon no bitoku* [The Virtues of Japan],” said, “since obtaining Japanese citizenship, I have come to think that I should …express my opinions as a Japanese citizen,” and, “now that I have become a Japanese citizen, I intend to freely speak out against Japan in ways that I have refrained from in the past.”

There can be no deep-rooted democracy in a society where the act of criticism itself comes under critique even before validation of the substance of the criticism. In Japanese society today, however, many people internalize an obedient spirit of servility toward current systems, without so much as questioning authority.

I too have been quite hesitant to criticize Japan up to this point, but like Keene, I must say what should be said before I reach the final years of my life. In this book, I speak of what I call “the dark side” of Japan, as seen through the lens of bioethics, so that non-Japanese readers can truly understand Japan. There are already enough publications which describe Japan’s advantages.
